# Pharmacokinetic Comparison of Berberine in Rat Plasma after Oral Administration of Berberine Hydrochloride in Normal and Post Inflammation Irritable Bowel Syndrome Rats

**DOI:** 10.3390/ijms15010456

**Published:** 2014-01-02

**Authors:** Zipeng Gong, Ying Chen, Ruijie Zhang, Yinghan Wang, Yan Guo, Qing Yang, Haixian Zhang, Yu Dong, Xiaogang Weng, Shuangrong Gao, Xiaoxin Zhu

**Affiliations:** 1 Institute of Chinese Materia Medica, China Academy of Chinese Medical Sciences, No.16, Dongzhimen Nei Nanxiao Road, Dongcheng District, Beijing 100700, China; E-Mails: gzp4012607@126.com (Z.G.); chenying0919@hotmail.com (Y.C.); zoucheng1218@163.com (R.Z.); wyh811018@163.com (Y.W.); yaoxueguoyan@163.com (Y.G.); yangqing121328@aliyun.com (Q.Y.); xgweng75@126.com (X.W.); rdou8@sohu.com (S.G.); 2 Guang’an Men Hospital, China Academy of Chinese Medical Sciences, No.5, Beixiange Road, Xicheng District, Beijing 100053, China; E-Mail: dongyu250541@sina.com; 3 Institute of Chinese Materia Medica, Chengde Medical University, Chengde 067000, China; 4 Institute of Ethnic Medicine, Southwest University, Chengdu 610041, China; E-Mail: 1986614haixian@163.com

**Keywords:** berberine, pharmacokinetic, absorption, irritable bowel syndrome

## Abstract

In the present study, post inflammation irritable bowel syndrome (PI-IBS) rats were firstly established by intracolonic instillation of acetic acid with restraint stress. Then the pharmacokinetics of berberine in the rat plasma were compared after oral administration of berberine hydrochloride (25 mg/kg) to normal rats and PI-IBS rats. Quantification of berberine in the rat plasma was achieved by using a sensitive and rapid UPLC-MS/MS method. Plasma samples were collected at 15 different points in time and the pharmacokinetic parameters were analyzed by WinNonlin software. Compared with the normal group, area under the plasma concentration *vs.* time curve from zero to last sampling time (*AUC*_0–t_) and total body clearance (*CL/F*) in the model group significantly increased or decreased, (2039.49 ± 492.24 *vs.* 2763.43 ± 203.14; 4999.34 ± 1198.79 *vs.* 3270.57 ± 58.32) respectively. The results indicated that the pharmacokinetic process of berberine could be altered in PI-IBS pathological conditions.

## 1. Introduction

Berberine is a botanical alkaloid which is present in many plants and has been used for many years due to its inexpensiveness and low incidence of adverse effects [1]. It has been traditionally used as an antimicrobial agent initially, and achieved favorable therapeutic effects in treating diarrhea. Further research of its biological activities revealed a variety of other pharmacological properties and applications for treating chronic diseases including diabetes, hyperlipemia, cancer and hypertension [2–5]. According to clinical studies, berberine has a significant effect in the treatment of experimental colitis, diarrhea and irritable bowel syndrome [6–9].

It is reported that the underlying mechanisms of berberine-mediated antidiarrheal effects not only include the inhibition of growing bacteria and anti-inflammatory effects but also involve gut protection [10]. However, several investigators have only reported the pharmacokinetics of berberine among healthy animals and human [11–14] while the research of the pharmacokinetics of berberine in the irritable bowel syndrome model has not been touched upon up to now.

As we all know, drugs are used to treat diseases and only patients are the ultimate consumers of drugs. In recent years, more and more research shows that the pharmacokinetic parameter of drugs can be affected by the disease states [15–21]. It is possible that drug metabolic enzymes, transporters, cell membrane permeability and the change of microbe group could be affected by physiological and pathological changes, which enable the pharmacokinetics of drugs in the body to be altered, including the process of absorption, distribution, metabolism and excretion. Therefore, it is necessary and important to study the pharmacokinetics of drugs in the pathological state. In the present study, post inflammation irritable bowel syndrome (PI-IBS) rats were established by intracolonic instillation of acetic acid with restraint stress. Then the pharmacokinetics of berberine in rat plasma were compared after oral administration of berberine hydrochloride in normal and PI-IBS rats by UPLC-MS/MS methods.

## 2. Results

### 2.1. Recording of Distal Colonic Motility and Calculation of Motility Index (MI)

**Figure 1.**
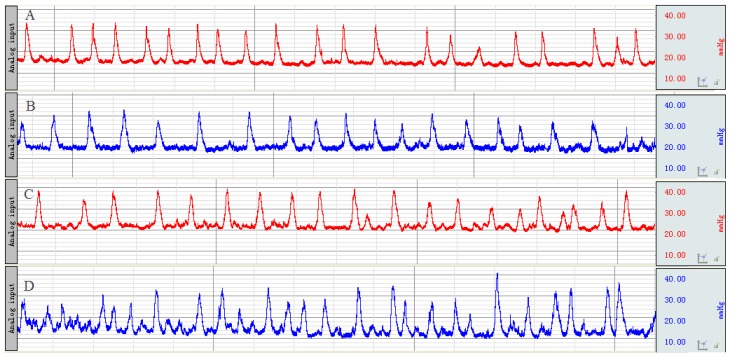
The representative curve of colonic movement in normal control group (**A**) and model group (**B**) before enema; in normal control group (**C**) and model group (**D**) after stress.

Before enema and after stress, the distal colonic MI was observed (Figure 1) and calculated (Table 1). At the time point before enema, there was no remarkable difference in MI between the two groups. After the stresses had been given, the distal colonic MI in the model group was significantly accelerated compared with the normal control group.

### 2.2. The Number of the Fecal Pellet Output over Two Hours

Before enema and after stress, the number of the fecal pellet output over 2 h was observed and calculated (Table 2). At the time point before enema, there was no remarkable difference in the number of the fecal pellet output between the two groups. However, the number of the fecal pellet output in the model group was significantly increased compared with the normal control group after the stresses had been given.

### 2.3. The Time of the Glass Bead Output

Before enema and after stress, the time of the glass bead output was observed and calculated (Table 3). At the time point before enema, there was no remarkable difference in the time of the glass bead output between the two groups. However, the time of the glass bead output in the model group was significantly shortened compared with the normal control group after the stresses had been given.

### 2.4. Histological Features of Colonic Tissue

**Figure 2.**
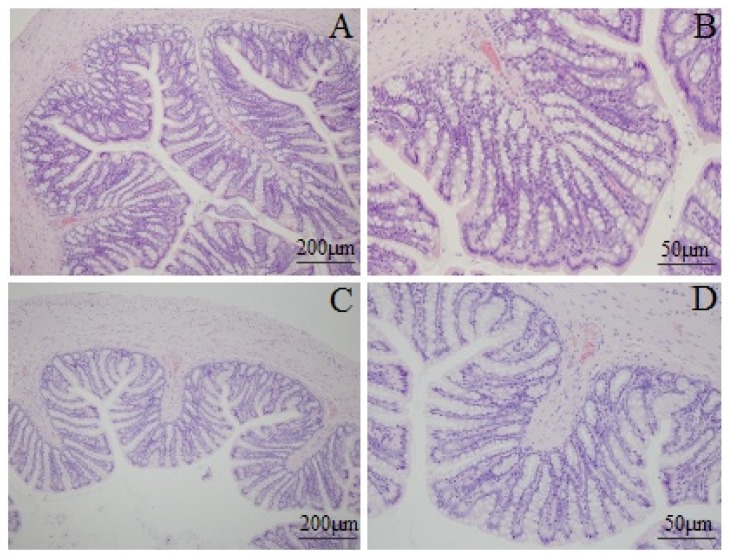
Photomicrographs of distal colons from the normal control group (**A**, 100×; **B**, 400×) and model group (**C**, 100×; **D**, 400×) by hematoxylin and eosin staining.

Mucosal histological features in the lamina propria and the submucosa were observed with an Olympus microscope. Figure 2 shows the structure of the colonic mucosa was clear with integrity, including a continuous and integral intestinal epithelium, regular glandular arrangement and no abnormal cells. In addition, little inflammatory cell infiltration was seen in the lamina propria. There was no remarkable inflammatory feature in the colon of the rats in the normal and the model group.

### 2.5. Mast Cell Count in Proximal Colon

**Figure 3.**
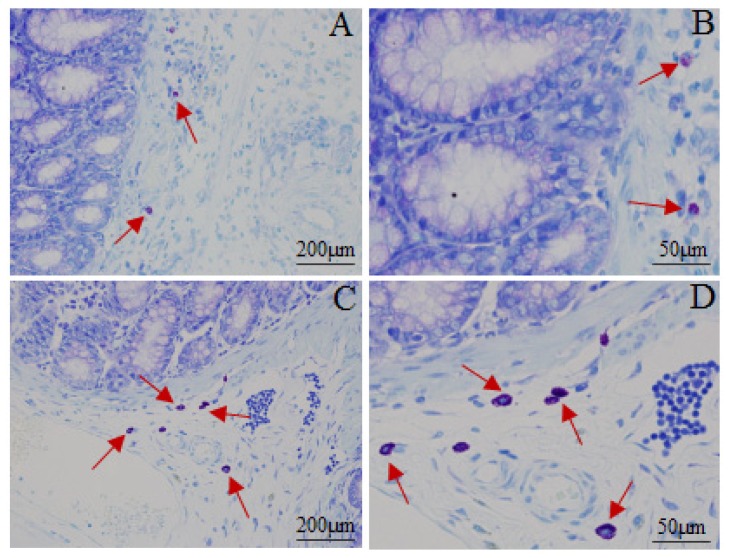
Photomicrographs of mast cells in proximal colons from normal control group (**A**, 100×; **B**, 400×) and model group (**C**, 100×; **D**, 400×) by toluidine blue staining. The red arrows indicate the mast cells.

Figure 3 and Table 4 shows the distribution or quantity of the mast cells. Most of the mast cells were distributed in the submucosa and lamina propria by grouping, in line or around the vessel, lymphatic vessel and peripheral nerve. The mast cells were round, oval or irregular, featured as aubergine cytoplasm and blue karyon. Moreover, the smaller cells had less cytoplasm and clear periphery while the bigger ones not only had more cytoplasm and unclear peripheries but also had aubergine granules around the karyon. Distribution of the mast cells in the model group was the same as in the normal control group. The quantity of the mast cells in the model group increased remarkably. These results indicated that intracolonic instillation of acetic acid with restraint stress could cause anomaly of mast cells.

### 2.6. Pharmacokinetic Analysis

**Figure 4.**
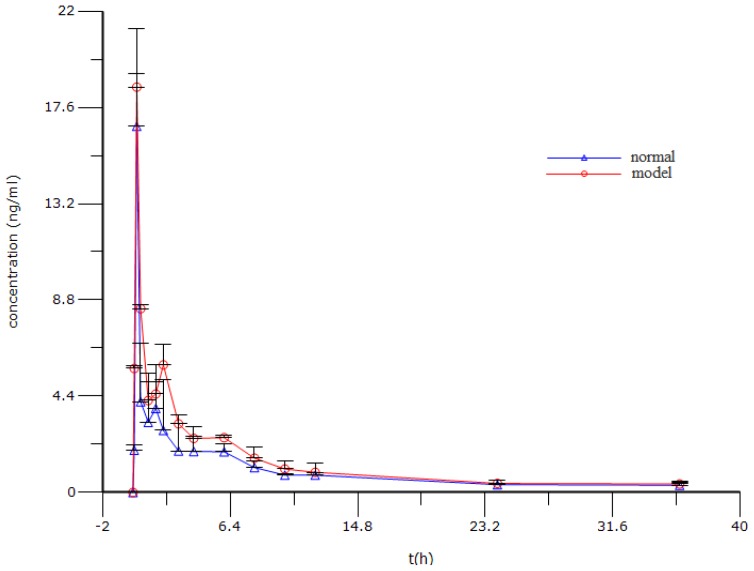
The mean plasma concentration (ng/mL) *vs.* time (h) profile after oral administration of berberine hydrochloride in the normal control and PI-IBS model rats. Values are expressed as mean ± SD (*n* = 5).

The mean plasma concentrations *vs.* time profiles of berberine following intragastric (i.g.) administration of berberine hydrochloride are shown in Figure 4 and its pharmacokinetic parameters are summarized in Table 5. The results show that the berberine was absorbed rapidly into the body 15 min after intragastric administration of berberine hydrochloride both in the normal control and the model group. Moreover, it is noteworthy that the size of the area under the plasma drug concentration time curves of berberine increased significantly in the model group (2763.43 ± 203.14) in comparison to the normal control group (2039.49 ± 492.24). Meanwhile, compared with that in the normal control group (4999.34 ± 1198.79), the marked decrease of *CL/F* of berberine in the model group (3270.57 ± 58.32) suggested that the elimination of berberine had slowed down.

## 3. Discussion

PI-IBS is a chronic functional digestive tract disease which is usually followed by acute infection of the gastrointestinal tract [22]. Clinical observations have found that the main symptoms of PI-IBS involve colonic dysfunction, abnormal defecation, abdominal pain and psychological symptoms including anxiety, depression, and so on. Until now, the pathogenesis of PI-IBS is still unclear although it is considered closely related to factors such as hereditary, society-psychological, pathogen, sex, age and abuse of antibiotics [23], which restrain the development of the proper PI-IBS animal model. Therefore, more attention was paid to the development of the proper PI-IBS animal model. In previous studies, the PI-IBS animal models were established by pathogens and chemicals, using stress to stimulate the animal inflammation. Although these models can partially imitate the symptoms of human PI-IBS, such PI-IBS models have respective disadvantages. For example, the models established by pathogens have poor repeatability and species specificity while inflammation caused by chemicals is far removed from human PI-IBS regarding pathogenesis [24]. In this study, PI-IBS rat models were established by intracolonic instillation of acetic acid to induce acute inflammation of the colon; after the inflammation resolved, wrap restraint stress was given to the rats. During the process of making the model, the rats in the model group had serious diarrhea in the first three days after enema which gradually resolved over the following days.

The mast cell is the natural ingredient of the neuroimmune axis and it can be activated to release a variety of bioactive substances including histamine, 5-HT, prostaglandin, leukotrienes and platelet-activating factor when stimulated by various kinds of physical and chemical factors or endogenous substances. More and more studies show that mast cells play an important role in the pathogenesis of IBS; the quantity and active degree of mast cells in the intestinal mucosa of IBS patients are abnormal [25]. Moreover, the number of mast cells increases more obviously in the intestinal mucosal of PI-IBS patients [26,27]. In the present study, similar results identified that the number of mast cells in the model group were dramatically increased.

Although the PI-IBS rat models in this study cannot avoid all the disadvantages of the traditional PI-IBS models, however restraint stress can not only significantly aggravate the colonic motility but also have a different influence on the function of the digestive tract and mast cell, which are the major syndromes of PI-IBS. These results point out that PI-IBS model rats established by intracolonic instillation of acetic acid with restraint stress can largely imitate the symptoms of human PI-IBS.

Berberine is a tablet of berberine hydrochloride which is used to treat infection of the digestive tract. Some clinicians have found berberine hydrochloride has a significant effect in the treatment of irritable bowel syndrome [28]. However, up to now, no attention has been paid to the pharmacokinetics of berberine in irritable bowel syndrome on animals and humans. Also, drugs are used to treat diseases and patients are the only ultimate consumers of drugs. Therefore, it is necessary to study the pharmacokinetics of berberine in the pathological state. Consequently, in our study, we compared the pharmacokinetics of berberine in rat plasma after dosage of oral berberine hydrochloride of 25mg/kg between normal and PI-IBS rats. The findings showed that *AUC*_(0–t)_ and *CL/F* in the model group significantly increased or decreased. Therefore, the results indicated that the pharmacokinetics of berberine in rat plasma were different between normal and PI-IBS rats. However, the mechanisms of the pharmacokinetic differences between normal and PI-IBS model rats were unclear, and this was possibly associated with the low inflammation in PI-IBS model rats. Some reports have shown that acute inflammation may alter the pharmacokinetics of a non-steroidal anti-inflammatory drug [29,30]. The discrepancy may come from the oscillation of the activities of cytochrome P450 (CYP) enzymes over the period of inflammation and drug taking.

In summary, these results support the conclusion that the pharmacokinetics of berberine in rat plasma could be altered in PI-IBS model rats in comparison to the normal rats. However, the extensional reason for the change is unclear and needs to be more deeply investigated.

## 4. Experimental Section

### 4.1. Materials

Berberine chloride with a purity of 86.7% was purchased from the National Institute for the Control of Pharmaceutical and Biological Products (Beijing, China). Methanol and acetonitrile of HPLC grade were obtained from Fisher Co. Ltd. (Waltham, MA, USA). Formic acid was obtained from Merck KGaACo. (Daemstadt, Germany). All other chemicals and reagents were analytical grade from Beijing Chemical Reagent Co. (Beijing, China). Milli-Q water (Millford, MA, USA) was used throughout the study.

### 4.2. Animals

Male Sprague-Dawley (SD) rats (230–270 g) were obtained from Beijing Vital River Laboratory Animal Technology Co. Ltd. (Beijing, China) and housed under standard conditions of temperature, humidity and light, and had free access to a standard rodent diet and water before the experiment. Animal welfare and experimental procedures were strictly in accordance with the Guide for the Care and Use of Laboratory Animals (US National Research Council, 1996). The animal protocol was approved by the Animal Ethics Committee at the Institute of Chinese Materia Medica, China Academy of Chinese Medical Sciences. Animals were randomly divided into two groups including normal control and model group.

### 4.3. Induction of PI-IBS Rats

After an overnight fast, acute colonic inflammation of rats in the model group was induced by intracolonic instillation of 1 mL 4% acetic acid by a silicone tube connected with injector at 8 cm proximal to the anus for 30 s. Then, 1 mL phosphate buffered saline was instilled to dilute the acetic acid and rinse the colon. Normal control animals were handled identically except that 1 mL saline was instilled instead of 4% acetic acid [31]. After 7 days post-enema, the rats in the model group were wrapped; the front upper limb, chest and front porch by adhesive tape for 1 h [32].

#### 4.3.1. Recording of Distal Colonic Motility and Calculation of Motility Index

After an overnight fast, the rats were put in a rubber capsule containing water which was connected to the recording system [33] (BIOPAC, MP150, physiograph; BIOPAC, Goleta, CA, USA) at 8 cm proximal to the anus. Colonic motility was recorded twice for 30 min including before enema and after stress.

Quantification of colonic motility was studied by calculating the motility index (MI). The MI was equivalent to the area under the curve of motility recording and was calculated by a computer-assisted system (BIOPAC systems, Inc, Goleta, CA, USA) [34].

#### 4.3.2. Measurement of the Number of Feces Defecated in 2 h and the Time of Glass Bead Output

After measuring the colonic motility of rats, glass beads of a diameter of 3 mm were put into the rectum along the anus which is 3 cm from the anus. Afterwards, the rats were put into the cage with clean filter pads and they had free access to standard rodent diet and water. Then, the number of feces defecated in 2 h and the time of glass bead output were measured.

#### 4.3.3. Histological Examination of Inflammation and Mast Cells Counting

To examine the mast cell and the extent of colonic inflammation, the proximal and distal colon were collected and sections with a thickness of 4 μm were cut and processed for toluene ammonia blue and hematoxylin-eosin staining. The coded slides were analyzed by Shuangrong Gao, a pathologist in the laboratory of the China Academy of Chinese Medical Sciences and the samples were blinded with regard to the drug group. The number of mast cells was counted in a six high power field (400×) for each coded slide and the mean number of mast cells were calculated.

### 4.4. Drug Analysis

Plasma concentrations of berberine were determined by using the UPLC-MS/MS method previously developed and validated [35]. Briefly, diphenhydramine was used as internal standard (IS). Quantitation was performed using multiple-reaction monitoring (MRM) of the protonated molecular ion to predominant product ion pair, *m*/*z* 336 > 320 for berberine and *m*/*z* 256 > 152 for IS. An aliquot of 100 μL plasma sample was spiked with 20 μL of the IS working solution of 100 ng/mL and vortexed briefly. Then the mixture was added to 360 μL of acetonitrile to be deproteinized, mixed by vortex for 5 min and centrifuged at 13,000 rpm for 15 min at 4 °C. The supernatant was evaporated by a gentle stream of nitrogen gas. The residue was reconstituted in 200 μL of the mobile phase followed by centrifugation at 13,000 rpm for 15 min. The supernatant was transferred into an autosampler vial and an aliquot of 3 μL was subsequently injected into the UPLC-MS/MS system for assay. The lower limit of quantification was established at 0.32 ng/mL for berberine in plasma.

### 4.5. Pharmacokinetic Analysis

Before the experiment, the rat was fasted overnight and then subjected to the following surgery under anesthesia, condition by intraperitoneal injection of chloral hydrate at 150 mg/kg. A polyethylene catheter (0.50 mm i.d., 1.00 mm o.d., Portex Limited, Hythe, UK) was cannulated into the right jugular vein. The distal end of the catheter was led under the skin and exteriorized at the back of the neck. After surgery, the rat was then allowed to recover for 24 h and fasted overnight prior to drug administration. Berberine hydrochloride freshly prepared in pure water with the aid of an ultrasonic instrument was i.g. administered to rats. After drug administration, the blood samples (200 μL) were collected from the catheter into heparinized centrifuge tubes at appropriate intervals (0.0833, 0.25, 0.5, 1, 1.5, 2, 3, 4, 6, 8, 10, 12, 24, 36 h). After centrifugation at 3500 rpm for 15 min, 100 μL of plasma was collected and stored at −80 °C until analysis. After each blood collection, 200 μL of normal saline containing 20 units/mL of heparin was immediately injected back into the body to flush the catheter and prevent coagulation. The amounts of berberine in the plasma were estimated by UPLC-MS/MS analysis as described previously.

### 4.6. Pharmacokinetic Data Analysis

The plasma concentrations *vs.* time profiles were analyzed using WinNonlin software (Version 6.3, Pharsight Corporation, Mountain View, CA, USA). The non-compartmental model was employed to estimate the following pharmacokinetic parameters: terminal elimination half-life (*t*_1/2_, λ_z_), area under the plasma concentration *vs.* time curve from zero to last sampling time (*AUC*_0–t_), volume of distribution (*V*_d_, λ_z_), total body clearance (*CL*). The peak plasma concentration (*C*_max_) and the time to reach *C*_max_ (*T*_max_) for the i.g. dose were read directly from the observed individual plasma concentration-time data.

### 4.7. Data Analysis

All reported values represent mean ± SD. The statistical difference was calculated using an unpaired *t*-test with a two-tailed distribution for comparison of two mean values. A *p* value < 0.05 was considered statistically significant.

## 5. Conclusions

The pharmacokinetic process of berberine can be altered in PI-IBS pathological conditions. The difference in the pharmacokinetic processes of berberine between normal and PI-IBS pathological conditions can help us explain and predict a variety of events related to the efficacy and toxicity of berberine.

## Figures and Tables

**Table 1.** The distal colonic motility index (MI) of rats (mmHg·s) (mean ± SD, *n* = 10).

Group	Before enema	After stress
Normal	1085.57 ± 134.93	1096.29 ± 119.53
Model	1098.86 ± 150.18	2107.29 ± 270.30 **

** *p* < 0.01 compared with normal group.

**Table 2.** The number of the fecal pellet output over 2 h (piece) (mean ± SD, *n* = 10).

Group	Before enema	After stress
Normal	4.43 ± 0.98	5.14 ± 1.07
Model	4.57 ± 1.13	8.29 ± 1.11 **

** *p* < 0.01 compared with normal group.

**Table 3.** The time of the glass bead output(s) (mean ± SD, *n* = 10).

Group	Before enema	After stress
Normal	1837.71 ± 160.54	1859.14 ± 102.08
Model	1772.57 ± 227.97	1297.71 ± 139.76 **

** *p* < 0.01 compared with normal group.

**Table 4.** The number of mast cells in the proximal colon (piece) (mean ± SD, *n* = 5).

Group	Mast cell count after stress
Normal	2.27 ± 1.05
Model	6.08 ± 2.28 **

** *p* < 0.01 compared with normal group.

**Table 5.** Pharmacokinetic parameters of berberine in rats after intragastric (i.g.) administration (mean ± SD, *n* = 5).

Parameters	Normal	Model
*T*_1/2,λz_ (min)	770.36 ± 65.01	941.45 ± 60.39
*T*_max_ (min)	15.00 ± 0.00	15.00 ± 0.00
*C*_max_ (ng/mL)	16.74 ± 4.47	18.53 ± 0.61
*AUC*_0–t_ (min ng/mL)	2,039.49 ± 492.24	2,763.43 ± 203.14 *
*V*_d_/*F*_λz_ (L/kg)	60,036.51 ± 19,704.59	41,202.89 ± 4,112.68
*CL/F* (L/h/kg)	4,999.34 ± 1,198.79	3,270.57 ± 58.32 *

* *p* < 0.05 compared with normal group.
